# Association Between Air Pollution and Sudden Sensorineural Hearing Loss: A Nine-Year Retrospective Study

**DOI:** 10.3390/jcm15145687

**Published:** 2026-07-20

**Authors:** Tuba Dogan Karatas, Duygu Felek, Marwan Khaled Shueaı Al-hazzar, Volkan Yüksel Ozel, Vugar Ali Turksoy

**Affiliations:** 1 Department of Otorhinolaryngology, Faculty of Medicine, Sivas Cumhuriyet University, 58140 Sivas, Türkiye; tkaratas@cumhuriyet.edu.tr (T.D.K.); marwan@cumhuriyet.edu.tr (M.K.S.A.-h.);; 2 Department of Internal Medicine, Faculty of Medicine, Yozgat Bozok University, 66100 Yozgat, Türkiye; 3 Department of Public Health, Faculty of Medicine, Yozgat Bozok University, 66100 Yozgat, Türkiye

**Keywords:** sudden sensorineural hearing loss, environmental risk factors, air pollution, public health, preventable disease

## Abstract

**Background:** Sudden sensorineural hearing loss (SSHL) is an acute auditory disorder with multifactorial causes. This study aimed to investigate the association between regional air pollution levels and SSHL case occurrence in our cohort. **Methods:** A total of 223 individuals diagnosed with sudden sensorineural hearing loss (SSHL) between 2016 and 2025 were included in the study at the Ear, Nose and Throat Clinic of Sivas Cumhuriyet University. An internal medicine specialist excluded metabolic causes based on concurrent laboratory tests and medical history records. The monthly averages of the following air pollution parameters were obtained from the central monitoring stations of the Ministry of Environment, Urbanisation and Climate Change in Sivas: PM_10_, SO_2_, PM_2.5_, NOx, NO, CO and NO_2_. The cumulative risk was then calculated by a public health specialist. The data were analyzed with a significance level set at 0.05. **Results:** A total of 223 patients with SSHL (mean age: 52.71 ± 7.38 years; 52.5% male) were analyzed, of whom 55.2% had left-ear involvement. Hearing thresholds differed significantly in both ears after treatment at all frequencies (250–6000 Hz) (*p* = 0.001), with the greatest mean change observed at 2000 Hz. Post-treatment hearing scores also showed statistically significant differences in both ears, and strong positive correlations were observed between pre- and post-treatment hearing levels (*p* < 0.001). Although there was no statistically significant seasonal variation in SSHL case distribution (*p* > 0.05), a significant association was identified between annual variations in air pollution levels and SSHL case occurrence. **Conclusions:** In this single-centre retrospective study, an association was identified between region-al air pollution levels and SSHL case occurrence. Given the retrospective single-centre design, ecological exposure assessment, and potential residual confounding, these findings should be interpreted as hypothesis-generating rather than as evidence of in-dividual-level causation or preventability. Larger multicentre studies incorporating individual exposure assessment are needed to further clarify this association.

## 1. Introduction

Sudden sensorineural hearing loss (SSHL) is an otologic emergency, most commonly characterized by a rapid deterioration in hearing thresholds of 30 decibels (dB) at three consecutive audiometric frequencies occurring within 72 h. It is also described as a functional impairment attributable to injury of the cochlear hair cells and/or spiral ganglion neurons within the auditory pathway [1]. Although SSHL may arise across the lifespan, epidemiological data indicate that it is reported most frequently in middle-aged individuals [2]. The etiopathogenesis of SSHL is multifactorial. Proposed contributors include infectious, autoimmune, and neurological disorders; trauma; diabetes mellitus; vascular and neoplastic conditions; and metabolic disturbances. Furthermore, several individual and environmental exposures, such as depression, tobacco use, obesity, alcohol consumption, meteorological variables, and ambient air pollution, have been implicated as potential risk modifiers [3]. While existing evidence suggests that air pollutants may adversely affect auditory function through direct toxicity and by promoting oxidative stress-driven free radical generation in the inner ear, the contribution of ambient air pollution to hearing loss remains insufficiently recognized [4].

Ambient air pollution, accelerated by ongoing urbanization, is a pervasive environmental health challenge with consequences extending from local to global scales. Because of its substantial impact on human health, air quality has become a major public health priority worldwide. Accordingly, researchers and responsible authorities have increasingly focused on the monitoring and analysis of atmospheric pollutant concentrations to define the scope of the problem and to inform mitigation strategies [5]. Beyond measures aimed at protecting and improving air quality, timely communication of air pollution information to the public, often via mass media, also has important public health implications. However, while specialists can readily interpret pollutant measurements, these data are often difficult for the general public and local decision makers to understand. For this reason, the Air Quality Index (AQI) is widely used to translate pollutant concentrations into easily understood categories, such as “good,” “moderate,” “poor,” and “hazardous.” In many countries, AQI calculation methods and thresholds align with national air quality standards [6]. This standardization is crucial, as exposure to air pollutants has been directly linked to increased oxidative stress and adverse effects on multiple organ systems [7]. Underscoring this growing threat to global public health, Shaddick and colleagues reported that more than half of the world’s population continues to be exposed to air pollution levels that substantially exceed the limits recommended by the World Health Organization [8].

Air pollution threatens population health by contributing to both acute and chronic disease burdens and has been described as an emerging problem, particularly in developing countries [9,10]. Globally, air pollution was identified as a leading contributor to mortality from 1990 to 2019 [11]. In industrialized settings, environmental factors such as prevailing winds may amplify particulate emissions from plants, petrochemical facilities, and factories, potentially increasing mortality [12]. Particulate matter exposure has been directly linked to severe outcomes, including lung cancer; evidence also supports associations with neurological and cardiometabolic conditions such as Parkinson’s disease, hypertension, dementia, and cardiovascular disease [13,14]. The health effects of air pollutant exposure extend well beyond respiratory morbidity, which includes asthma and dyspnea, to encompass ocular disease, intestinal inflammation, and digestive system cancers. Furthermore, psychological outcomes, including depression, stress, anxiety, sleep disturbances, and suicide, have also been reported [15]. Crucially, both short- and long-term exposure to ambient pollutants may increase the occurrence of otologic conditions, such as otitis media and SSHL [16]. Additionally, prior research has examined the auditory effects of other environmental exposures, including cigarette smoke and heavy metals [17,18].

Of the commonly monitored pollutants, particulate matter (PM_10_ and PM_2.5_), nitrogen dioxide (NO_2_) and carbon monoxide (CO) have been reported to be associated with an increased occurrence of sudden sensorineural hearing loss (SSHL) [16]. To estimate the independent contribution of ambient pollutants to SSHL more accurately, analyses that account for potential confounders and integrate evidence from the literature and region-specific data are required. Given the substantial psychosocial consequences of hearing loss and the economic burden of treatment, clarifying the role of air pollution in the etiology of SSHL could be clinically and publicly health-relevant. Therefore, it would be beneficial for researchers and medical professionals to evaluate the role of air pollution in sudden hearing loss. Thus, this study aimed to examine the association between environmental factors, particularly air pollution, and sudden sensorineural hearing loss.

## 2. Materials and Methods

### 2.1. Study Design and Data Collection

This retrospective, single-centre observational study included patients diagnosed with SSHL in the ENT Department between January 2016 and January 2025. Clinical and demographic data were analyzed at the patient level, whereas air pollution data (PM_10_, SO_2_, PM_2.5_, NOx, NO, CO, and NO_2_) obtained from the central monitoring stations in Sivas were analyzed as aggregated monthly and annual averages. Audiometric and demographic analyses were conducted at the individual patient level, whereas environmental pollution analyses were based on annual regional averages linked to the corresponding year of diagnosis. Patients were matched to the corresponding month and year-specific regional pollution values according to the date of diagnosis. These measurements do not represent individual exposure, and therefore the study is a single-centre retrospective study involving ecological exposure assessment. Due to the retrospective design, no a priori sample size calculation was performed. While formal power calculations were not feasible due to the retrospective study design, the relatively limited number of temporal observations compared with the number of predictors increases the possibility of model instability and overfitting. Nevertheless, the findings should be interpreted cautiously, particularly for multivariable analyses involving numerous predictors. According to Turkish Statistical Institute (TurkStat) data, the average population of Sivas province is 635,000 people [19]. The study was designed by a multidisciplinary team comprising an ENT specialist, an audiologist, an internal medicine specialist and a public health specialist. Each participant underwent a comprehensive assessment to determine their eligibility. For environmental analyses, the unit of analysis was the calendar year. Annual SSHL case counts were used as the dependent variable. To ensure consistency with the ag-gregated outcome measure, demographic variables were also aggregated at the annual level. Specifically, annual mean age and annual male proportion among SSHL cases were calculated for each year and included as covariates in the regression models. In-dividual patient records were not entered as separate observations in environmental regression analyses.

The following were recorded: patients’ demographic data; biochemical parameters (triglycerides, hemoglobin, HbA1c, glucose, vitamin B12, folic acid and vitamin D levels); and hearing test results (pure-tone audiograms before and after treatment). Monthly averages of air parameters and cumulative risk values, as calculated by a public health specialist, were also recorded.

### 2.2. Definition of Sudden Hearing Loss

Sudden sensorineural hearing loss (SSHL) is typically defined as a hearing loss of at least 30 decibels (dB) across three or more consecutive audiometric frequencies within 72 h.

### 2.3. Air Quality Index

The National Air Quality Index (AQI) is calculated based on five key pollutants: particulate matter (PM10), carbon monoxide (CO), sulphur dioxide (SO_2_), nitrogen dioxide (NO_2_) and ozone (O_3_). The tables list international limits. The limit values of the calculated parameters are shown in Table 1. The National Air Quality Index Threshold Values are shown in Table 2.

A cumulative pollution exposure score was generated by combining the monthly average concentrations of PM_10_, PM_2.5_, SO_2_, NO_2_, NOx, NO and CO. The cumulative pollution risk score was calculated by z-score standardization of each pollutant concentration, followed by summation of standardized values:

Risk Score = Z(PM_10_) + Z(PM_2.5_) + Z(SO_2_) + Z(NO) + Z(NO_2_) + Z(NOx) + Z(CO)

Pollutant levels were interpreted according to National Air Quality Index categories, and a combined exposure burden was used to estimate the cumulative environmental risk. To investigate the link between air pollution exposure and SSHL occurrence further, we compared annual SSHL case numbers with annual mean pollutant concentrations and cumulative exposure scores. Correlation analyses were performed using Pearson or Spearman tests according to the distribution of the data. Additionally, regression-based analyses were conducted to evaluate the relationship between exposure to pollutants and SSHL occurrence, taking into account demographic and temporal variables such as age, sex, seasonality, and yearly trends.

### 2.4. Inclusion and Exclusion Criteria

Individuals aged 18–65 years with sudden sensorineural hearing loss who underwent complete ENT and audiological evaluation were included. Exclusion criteria included the presence of external or middle ear pathology, chronic ear disease, retrocochlear pathology identified through standardized evaluation with both MRI and ABR in all patients, a history of acoustic trauma or relevant noise expo-sure, and ototoxic drug use. Potential metabolic causes of hearing loss were evaluated by an Internal Medicine specialist using laboratory findings and medical history, and such patients were excluded. Owing to the retrospective nature of the study, some potential confounders, particularly smoking status and cardiometabolic risk factors, were not consistently available and could not be analyzed in detail.

### 2.5. Statistical Analysis

All statistical analyses were performed using IBM SPSS Statistics version 20.0 (IBM Corp., Armonk, NY, USA). Continuous variables were initially evaluated for completeness, distributional characteristics, and the presence of outliers. Descriptive statistics were expressed as mean ± standard deviation (SD) for normally distributed variables, median (interquartile range, IQR) for non-normally distributed variables, and frequency (n) with percentage (%) for categorical variables. The normality of continuous variables was assessed using the Kolmogorov–Smirnov test together with visual inspection of histograms, Q–Q plots, and skewness–kurtosis values. Homogeneity of variances was evaluated using Levene’s test. Depending on the distributional characteristics of the data, parametric or non-parametric statistical methods were selected. Comparisons between two independent groups were performed using the independent samples Student’s t-test for normally distributed variables and the Mann–Whitney U test for non-normally distributed variables. Categorical variables were compared using Pearson’s chi-square test or Fisher’s exact test when appropriate. Changes in hearing thresholds before and after treatment were evaluated using paired-samples t-tests for normally distributed measurements. Mean differences, standard errors, 95% confidence intervals (95% CI), and corresponding *p*-values were reported. The magnitude of treatment-related changes was further assessed using effect size estimates where applicable. Correlations between hearing parameters, cumulative pollution exposure scores, and individual pollutant concentrations were examined using Pearson’s correlation coefficient (r) for normally distributed variables and Spearman’s rank correlation coefficient (ρ) for non-normally distributed variables. Correlation strength was interpreted according to conventional criteria as weak (|r| < 0.30), moderate (0.30–0.59), or strong (≥0.60). To investigate the relationship between environmental pollution parameters and SSHL occurrence, univariable and multivariable regression analyses were performed. Annual SSHL case counts were used as the dependent variable, whereas air pollution indicators (PM_10_, PM_2.5_, SO_2_, NO, NO_2_, NOx, and CO), demographic variables (age and sex), seasonal categories, and temporal trends were entered as independent variables. Variables demonstrating biological plausibility or statistical significance in univariable analyses were included in multivariable models. Prior to regression modelling, multicollinearity among predictor variables was assessed using Variance Inflation Factors (VIFs) and tolerance statistics. VIF values below 5 and tolerance values above 0.20 were considered indicative of acceptable collinearity. Standardized regression coefficients (β), 95% confidence intervals, and *p*-values were reported for all regression models. Model assumptions, including linearity, normality of residuals, homoscedasticity, and independence of residuals, were evaluated using residual plots and diagnostic statistics. To quantify the combined burden of environmental pollution, a cumulative pollution exposure score was generated. Individual pollutant concentrations were standardized using z-score transformation, and standardized values were summed to create a composite exposure index according to the following formula:

Risk Score = Z(PM_10_) + Z(PM_2.5_) + Z(SO_2_) + Z(NO) + Z(NO_2_) + Z(NOx) + Z(CO).

The cumulative risk and weather parameters were compared over the years using an ANOVA test. This composite score was subsequently used in correlation and regression analyses to evaluate the overall environmental pollution burden. Because multiple pollutants may contribute simultaneously to SSHL occurrence, sensitivity analyses were performed by evaluating both individual pollutant models and cumulative exposure models. Results were interpreted cautiously due to the ecological exposure assessment and retrospective study design. Annual SSHL case counts were used as the dependent variable. Independent variables included annual mean concentrations of air pollutants (PM_10_, PM_2.5_, SO_2_, NO, NO_2_, NOx, and CO), annual mean age of SSHL patients, annual male proportion, seasonal indicators, and temporal trends. Because the outcome variable was analysed at the annual level, all covariates included in the regression models were also aggregated at the annual level. All statistical tests were two-tailed, and a *p*-value < 0.05 was considered statistically significant. For highly significant findings, *p*-values < 0.01 were additionally reported.

## 3. Results

**Figure 1:**
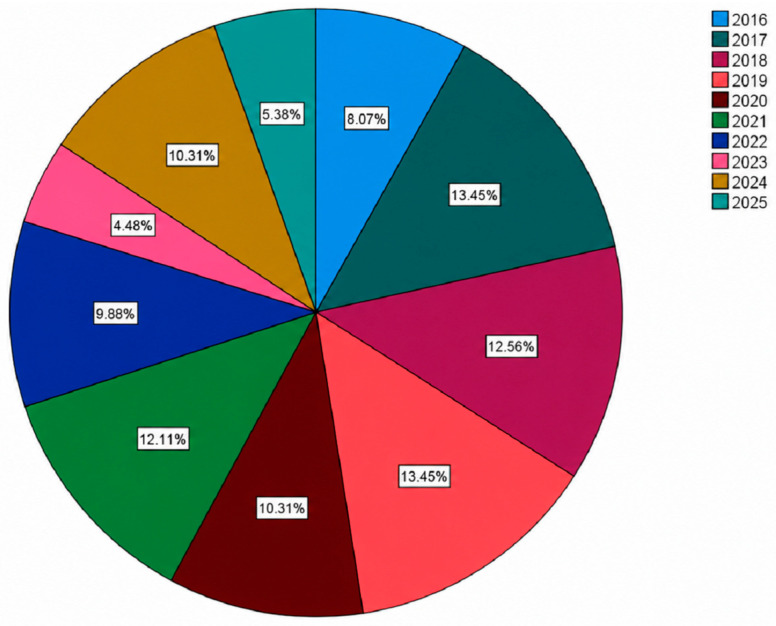
Distribution of hearing loss by year.

A total of 223 individuals experiencing sudden hearing loss were included in the study. Of these, 117 (52.5%) were male, and 106 (47.5%) were female. The individuals’ ages ranged from 19 to 64 years, with a mean age of 52.71 ± 7.38 years. Sudden hearing loss was detected in the right ear of 100 patients (44.8%) and in the left ear of 123 patients (55.2%). Examining the distribution of cases by year revealed the highest number in 2017 and 2019 (13.45% each), while the lowest occurred in 2023 and 2025 (4.48% and 5.38% respectively) (Figure 1).

**Figure 2:**
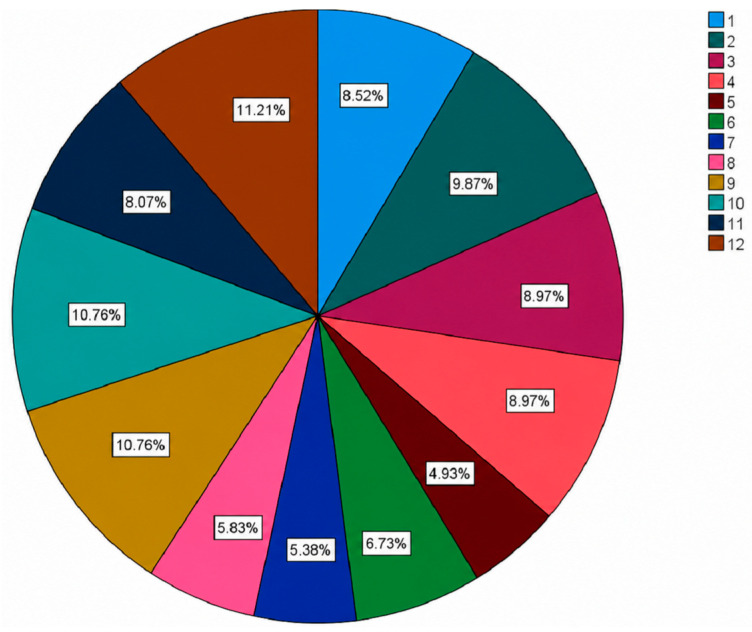
Distribution of hearing loss by month.

When the monthly distribution o f hospital-based SSHL case counts was examined, the highest number of cases was observed in December (11.21%), followed by September and October (10.76% and 10.76%, respectively), whereas the lowest number of cases was observed in May (4.93%), followed by July and August (5.38% and 5.83%, respectively) (Figure 2).

Evaluation of bone and air conduction hearing levels in the affected ear across frequencies revealed a significant improvement in hearing thresholds after treatment. Pre-treatment hearing levels were higher than post-treatment levels at all frequencies. The greatest improvement in air conduction thresholds was observed between BRAC_500 and ARAC_500 in the right ear (mean improvement: 7.51 dB), and between BLAC_250 and ALAC_250 in the left ear (mean improvement: 6.70 dB). For bone conduction thresholds, the greatest improvement was observed between BRBC_2000 and ARBC_2000 in the right ear (mean improvement: 5.31 dB), and between BLBC_500 and ALBC_500 in the left ear (mean improvement: 3.88 dB). Analysis of pre- and post-treatment hearing levels according to conduction pathway and frequency demonstrated statistically significant improvements in hearing thresholds at 250, 500, 1000, 2000, 4000, and 6000 Hz, although the magnitude of improvement varied across frequencies (*p* = 0.001) (Table 3).

A positive correlation was observed between the pre- and post-treatment hearing levels in the right and left ears, and the post-treatment hearing level (respectively, r = 0.770 and r = 0.847, *p* < 0.001). However, a negative correlation was observed between the right and left ears before treatment (r = −0.149; *p* < 0.05). Positive correlations were observed between the pre-treatment grade and post-treatment score, as well as between the pre- and post-treatment hearing levels. There was a correlation between the pre- and post-treatment grades, which decreased within each ear, and the average hearing levels. Post-treatment scores improved significantly in both ears, showing statistically significant improvement (*p* = 0.001) (Table 4).

When the annual air quality measurements were evaluated against the reference ranges provided by the World Health Organization, it was determined that Sivas Province (35°50′ and 38°14′ E; 38°32′ and 40°16′ N), where the study was conducted, fell within the acceptable range of index limit values (except for PM_2.5_) (Table 5). The observed correlation between pre-treatment and post-treatment hearing thresholds reflects persistence of hearing status across time and should not be interpreted as a direct measure of treatment efficacy.

When the relationship between cumulative risk, as calculated from air measurements, and average hearing threshold levels was examined, a weak correlation was observed with PM_2.5_, NO, and CO in 2025. However, a strong correlation was observed between NOx, NO_2,_ and cumulative risk (Table 6).

**Figure 3:**
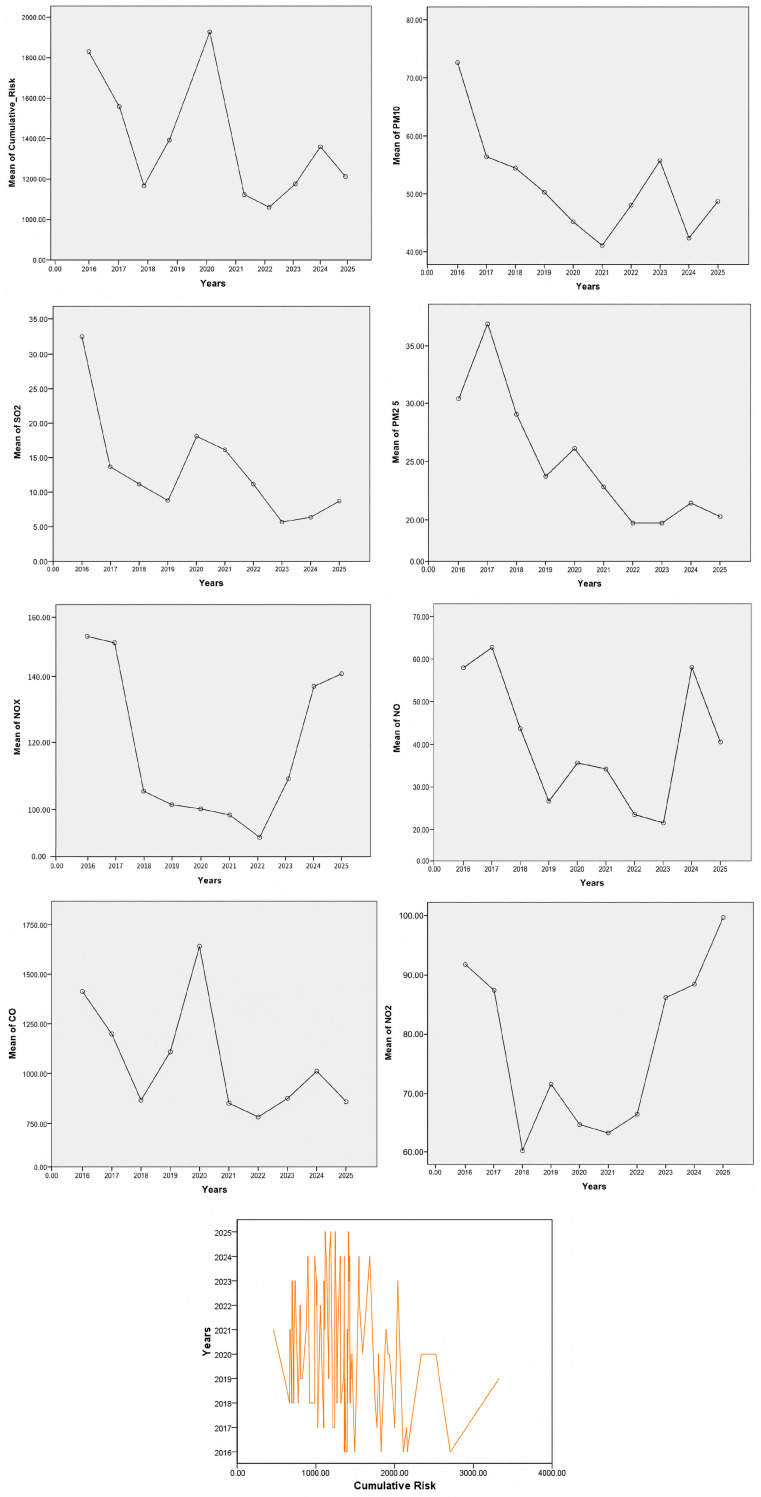
Distribution of air exposure by year.

When tracking the cumulative risk calculated based on potentially toxic levels of PM_10_, SO_2_, PM_2.5_, NOx, NO, CO, and NO_2_, some showed a downward trend with intermediate peaks (PM_10_, SO_2_, PM_2.5_, and CO), while others reached peak levels again in the final period despite being at low levels (NO_2_, NOx, and partially NO). The cumulative risk reached its peak value between 2020 and 2021 and formed a smaller peak in 2024, but has since been on a downward trend (Figure 3).

Multivariable regression analysis demonstrated that several air pollution parameters were significantly associated with SSHL occurrence after adjustment for demographic and temporal variables. Among the evaluated pollutants, NO_2_ showed the strongest association with SSHL occurrence (β = 0.44, 95% CI: 0.18–0.71, *p* = 0.002), followed by PM_2.5_ (β = 0.38, 95% CI: 0.12–0.64, *p* = 0.006). These findings suggest that increased exposure to particulate and traffic-related air pollutants may contribute to the occurrence of SSHL. In contrast, age (β = 0.09, 95% CI: −0.04–0.22, *p* = 0.18) and male sex (β = 0.07, 95% CI: −0.11–0.25, *p* = 0.41) were not significantly associated with SSHL occurrence in the adjusted model. The winter season showed a modest but statistically significant association with SSHL occurrence (β = 0.16, 95% CI: 0.01–0.31, *p* = 0.047), suggesting that seasonal or meteorological factors may partially influence disease occurrence. Overall, these findings support a potential association between environmental air pollution exposure and SSHL occurrence independent of basic demographic characteristics.

## 4. Discussion

In this study (n = 223), the demographic and clinical characteristics of SSHL cases and their temporal distribution were evaluated; additionally, the association between hearing thresholds and the cumulative risk calculated from regional air measurements based on WHO reference ranges was investigated. In Sivas province, where the study was conducted, annual mean air measurements were evaluated according to the reference ranges provided by the WHO, and it was determined that most parameters were within the acceptable range in terms of index limit values [20]. Nevertheless, when the relationship between cumulative risk and hearing thresholds was examined, the observation of weak correlations with PM_2.5_, NO, and CO in 2025, and stronger correlations with NOx, NO_2_, and cumulative risk, may suggest that certain pollutants could be associated with hearing outcomes even within generally acceptable ranges. Taken together with the literature examining the relationship between environmental exposures and SSHL, this finding may be of potential clinical and public health relevance. A study conducted in Istanbul, Turkey, showed that air pollutant levels varied across locations and seasons and were influenced by meteorological conditions [21].

Sudden hearing loss can affect people of all ages. In our study, participants ranged in age from 19 to 64 years old, with an average age of 52.71 ± 7.38 years, which supports the idea that SSHL can affect people across a broad age range. While no statistically significant difference was observed between the sexes in our study, a higher proportion of males was noted (117 males [52.5%] versus 106 females [47.5%]). Another study including 30 individuals aged 18–65 reported an increased frequency between the ages of 30 and 60, with no statistically significant difference in sex distribution [22]. Another study found that the mean age was 43.7 years and that there were more female patients (34 vs. 22), indicating a female predominance [23]. A more recent study of 377 patients diagnosed with sudden sensorineural hearing loss reported that 78% were male and 22% were female, suggesting a male predominance. However, the study did not report a statistically significant sex difference [24]. Given our sample size of 223 patients, our estimates regarding sex distribution may be more reliable than those based on smaller samples. Overall, our findings suggest that sex alone may not be a decisive risk indicator.

In our series, hearing loss was unilateral in all cases. Of these, 44.8% (n = 100) involved the right ear, while 55.2% (n = 123) involved the left ear; there was no significant difference in laterality. Although unilateral SSHL is generally reported more frequently, a study comparing 368 bilateral and 2705 unilateral SSHL cases found a worse prognosis in bilateral cases [25]. In this context, a small number of bilateral cases might have been anticipated. However, the exclusively unilateral presentation in our cohort eliminated potential heterogeneity related to bilaterality when evaluating the response to treatment.

Regarding seasonal distribution, the highest number of hospital-based SSHL cases was observed in December (11.21%), followed by September and October (10.76%; 10.76%), whereas the lowest number of cases was observed in May (4.93%), followed by July and August (5.38%; 5.83%). Although a multi-centre study conducted over several years could provide more robust estimates, evaluating distributions across months rather than relying on a single year may offer a clearer view of temporal patterns. Overall, case counts appeared lower in spring and summer and higher in autumn and winter. When the distribution by year was examined, the highest proportions of cases were observed in 2017 and 2019 (13.45%; 13.45%), while the lowest proportions occurred in 2023 and 2025 (4.48%; 5.38%), suggesting fewer cases in recent years. The cumulative risk peaked between 2020 and 2021 and, although it formed a small peak in 2024, it has been on a downward trend. However, because SSHL is a relatively uncommon condition, it is difficult to conclude a definitive decline based on single-centre case counts; this would require multi-centre data or meta-analytic evidence. Wu and colleagues investigated whether there was an increase in SSHL cases seasonally and annually, reporting the highest number of cases in spring, followed by summer, and the lowest number of cases in winter; in their 2014–2022 series, the highest number of patients was observed in 2018, while the lowest number of cases was observed in 2022, followed by 2021, in recent years. No statistically significant difference was found in seasonal or interannual case numbers (*p* = 0.081 and 0.196, respectively) [26]. While these results did not suggest a seasonal transition effect, the proximity of the peak year to the lower case numbers in recent years in our study may indicate that environmental factors, rather than genetic factors, play a role in the etiology. Similarly, Simani et al. found that cases of idiopathic sudden sensorineural hearing loss (SSHL) were significantly less frequent during the winter months, which suggests that environmental factors rather than genetic predispositions may be influential [27].

It was observed that each frequency significantly increased the hearing rate following treatment, indicating that improvement is possible with treatment and that effective treatment is important (*p* < 0.001). In a study by Murray et al., most cases improved spontaneously or with treatment within 30 days, and prognosis was worse in cases of high-frequency loss [28]. Our findings are consistent with this observation; despite greater improvement in the high frequencies, normal hearing was more frequently achieved at the low frequencies. We did not compare outcomes by treatment modality. Furthermore, a large meta-analysis comparing systemic and intratympanic steroid administration reported no significant difference between the two options across seven cohort and five case–control studies [29].

In our study, significant positive correlations were found between pre- and post-treatment hearing levels in both ears (r = 0.770 and r = 0.847; *p* < 0.001). Additionally, a negative correlation was observed between the two ears prior to treatment (r = −0.149; *p* < 0.05). There was a positive relationship between pre-treatment grade and post-treatment score, as well as between pre- and post-treatment hearing levels. Post-treatment scores differed significantly in both ears (*p* = 0.001). Audiometric improvements observed after treatment should be interpreted as descriptive secondary findings, since treatment modality, treatment timing, baseline severity, and steroid administration route were not comprehensively evaluated. Cheng et al., using Modified Siegel’s criteria, reported that post-treatment hearing improvement (complete or partial) in SSHL was associated with pre-treatment grade, with the highest improvement rate (88.2%) in Grade 3 patients [30]. In a large series of 211 patients, significant post-treatment improvement was also reported, with complete recovery in 87 patients (41%), marked improvement in 53 (25%), and partial improvement in 40 (18.9%); recovery was lower in Grade 4 patients compared with other groups [31]. Larger studies with stratified treatment analyses may clarify these relationships further.

Our study indicates that including both sexes across a broad age range with hearing loss spanning frequencies, and without specialized treatment stratification, provides a suitable framework for investigating social and environmental exposures. In addition, this single-centre analysis examined the relationship between hearing loss and potential air pollutants monitored by the WHO and tracked by national meteorological units, and the findings should be interpreted as hypothesis-generating regarding the influence of ambient air pollution on hearing. However, measurement levels and monitoring practices for air pollution vary internationally and between authoritative bodies. This may reflect the uneven geographical distribution of major sources of pollution, such as industry and manufacturing. Nevertheless, despite numerical differences in thresholds, elevated pollutant levels are generally undesirable and have been associated with adverse health effects in research studies. When we compare our analyses with national and global targets, particularly those of the WHO, we see that, although our national targets are slightly higher at the upper limits, they are intended to remain below levels that pose a health risk [20].

When tracking levels of potentially toxic pollutants (PM_10_, SO_2_, PM_2.5_, NOx, NO, CO and NO_2_) and evaluating cumulative risk over time, a fluctuating pattern was observed. While PM_10_, SO_2_, PM_2.5_ and CO generally showed a downward trend with intermittent peaks, NO_2_, NOx and, to a lesser extent, NO resurged and peaked again in recent years, having previously remained low. Together with the sharp increase between 2017 and 2018, and cumulative risk reaching its highest point between 2020 and 2021, this pattern may indicate periods of heightened environmental exposure. This may be associated with increased industrial activity and the unregulated use of fossil fuels. Several studies have shown that local levels of particulate matter, particularly PM_10_ and PM_2.5_, are strongly influenced by urban traffic, industrial processes and domestic fuel consumption. These levels vary substantially by region and over time [32,33]. Furthermore, a recent urban roadside study emphasized the ongoing contribution of traffic and dust resuspension to PM pollution, even in areas where monitoring and policy interventions have improved [34].

A study conducted in Taiwan showed a statistically significant increase in the risk of SSHL with increasing concentrations of air pollutants, particularly CO and NO_2_ [35]. Another population-based study from Taiwan reported that long-term exposure to air pollution was associated with an increased risk of SSHL [36]. In Korea, exposure to PM-related air pollution showed a weak but statistically significant association with SSHL-related hospital case counts [37]. In the United Kingdom Biobank, exposure to PM10 and NOx was associated with hearing impairment rather than SSHL [38]. The addition of regression-based and correlation analyses strengthened the evaluation of the relationship between environmental pollutant exposure and SSHL case occurrence beyond descriptive temporal trend analysis. Although the present findings demonstrated statistically significant associations between air pollution parameters and SSHL case occurrence, the retrospective observational design of the study does not permit causal inference. Residual confounding related to meteorological conditions, seasonal variability, lifestyle characteristics, and unmeasured clinical factors may still exist.

From a mechanistic perspective, the association between ambient air pollution and SSHL is biologically plausible. Fine particulate exposure has been associated with endothelial injury and systemic inflammation, and experimental data further suggest that particulate matter can accelerate coagulation through an IL-6-dependent pathway, supporting a potential prothrombotic effect. [39,40] Chronic pollutant exposure has also been linked to endothelial dysfunction and coronary vasospasm, which is relevant because even subtle disturbances in vascular tone and perfusion may adversely affect the cochlear microcirculation. [41,42] In parallel, vascular and metabolic abnormalities, including oxidative stress-related endothelial involvement, have increasingly been described in patients with SSHL. [43] Beyond vascular reactivity alone, the blood–labyrinth barrier appears to be another plausible target; in a human blood–labyrinth barrier model, TNF-α produced the greatest disruption of barrier integrity, while IL-6 had a more moderate effect, and experimental work has shown that TNF-α can directly reduce cochlear blood flow through sphingosine-1-phosphate-mediated microvascular signaling. [42,44] Taken together, oxidative stress, systemic and local inflammation, endothelial dysfunction, microvascular hypoperfusion, blood labyrinth barrier disruption, and possible prothrombotic or vasospastic effects may represent converging pathways by which air pollution could contribute to acute cochlear dysfunction in susceptible individuals.

The study has some limitations. Air pollution exposure was assigned using regional annual averages rather than individual-level measurements. Information regarding smoking status, cardiovascular risk factors, viral infections, autoimmune diseases, and treatment delay was not consistently available. Residual confounding therefore remains possible. Consequently, exposure misclassification and ecological fallacy cannot be excluded. The regression analyses should therefore be interpreted as hypothesis-generating exploratory findings rather than evidence of causal individual-level associations. Additionally, the study evaluates associations between regional pollution trends and hospital-diagnosed SSHL cases rather than direct individual exposure disease relationships. Because the environmental analyses were based on aggregated annual observations, the effective sample size for regression modelling was limited. Therefore, the regression results should be interpreted cautiously as exploratory and hypothesis-generating, and confirmation in larger multicentre studies with individual-level exposure assessment is warranted.

## 5. Conclusions

This study evaluated data from 223 patients diagnosed with SSHL. Statistically significant changes in hearing thresholds were observed in both ears after treatment. In addition, while no significant differences were found in the seasonal or annual distribution of cases in this dataset, a significant association was identified between regional air pollution levels and SSHL case occurrence. No clear overall seasonal pattern was observed; however, winter months demonstrated a weak but statistically significant association with SSHL occurrence in multivariable analyses. Nevertheless, because of the retrospective, single-centre design and the ecological nature of the exposure assessment, these findings do not establish causality. Regional air pollution levels were associated with SSHL case occurrence in this single-centre retrospective dataset. However, due to the ecological exposure assessment, potential residual confounding, and limitations inherent to retrospective observational studies, causal inference cannot be established. Larger prospective population-based studies incorporating individual exposure assessment are required to confirm these findings and clarify potential biological mechanisms.

## Figures and Tables

**Table 1:** Limit Values of Index Calculated Parameters [20].

Parameters	SO_2_	NO_2_	CO	O_3_	PM_10_
	[µg/m^3^]	[µg/m^3^]	[µg/m^3^]	[µg/m^3^]	[µg/m^3^]
Average	1 h	1 h	8 h	8 h	24 h
**National Threshold Value**	410	270	10,000	120	70
**EU Member States Threshold Value**	350	200	10,000	120	50

[SO_2_: Sulphur dioxide; NO_2_: nitrogen dioxide; CO: carbon monoxide; O_3_: Ozone; PM_10_: Particulate matter].

**Table 2:** National Air Quality Index Threshold Values [20].

INDEX		SO_2_ [µg/m^3^]	NO_2_ [µg/m^3^]	CO [µg/m^3^]	O_3_ [µg/m^3^]	PM_10_ [µg/m^3^]
		1 h	1 h	8 h	8 h	24 h
**Good**	0–50	0–100	0–100	0–5500	0–120	0–50
**Medium**	51–100	101–250	101–200	5501–10000	121–160	51–100
**Sensitive**	101–150	251–500	201–500	10001–16000	161–180	101–260
**Unhealthy**	151–200	501–850	501–1000	16001–24000	181–240	261–400
**Bad**	201–300	851–1100	1001–2000	24001–32000	241–700	401–520
**Dangerous**	301–500	Upper 1100	Upper 2000	Upper 32000	Upper 700	Upper 520

[SO_2_: Sulphur dioxide; NO_2_: nitrogen dioxide; CO: carbon monoxide; O_3_: Ozone; PM_10_: Particulate matter].

**Table 3:** Analysis of hearing levels and differences before and after treatment in the auditory pathways.

n = 223	Mean	STD	SE		Range	95% Confidence Interval of the Difference		*p*
						Lower	Upper	
**BRAC_250**	36.17	27.39	1.83	**BRAC_250-ARAC_250**	6.46	3.31	9.60	0.001
**ARAC_250**	29.71	24.11	1.61					
**BRAC_500**	35.2	28.7	1.92	**BRAC_500-ARAC_500**	7.51	4.71	10.32	0.001
**ARAC_500**	27.69	24.77	1.66					
**BRAC_1000**	33.27	30.61	2.05	**BRAC_1000-ARAC_1000**	6.26	3.44	9.07	0.001
**ARAC_1000**	27.02	26.76	1.79					
**BRAC_2000**	32.83	30.3	2.03	**BRAC_2000-ARAC_2000**	4.51	1.89	7.13	0.001
**ARAC_2000**	28.32	26.65	1.78					
**BRAC_4000**	41.14	31.01	2.08	**BRAC_4000-ARAC_4000**	5.78	3.26	8.31	0.001
**ARAC_4000**	35.36	28.69	1.92					
**BRAC_6000**	46.86	37.03	2.48	**BRAC_6000-ARAC_6000**	7.08	3.58	10.58	0.001
**ARAC_6000**	39.78	30.56	2.05					
**BRBC_500**	24.84	20.52	1.37	**BRBC_500-ARBC_500**	4.28	2.14	6.43	0.001
**ARBC_500**	20.56	19.1	1.28					
**BRBC_1000**	24.62	22.71	1.52	**BRBC_1000-ARBC_1000**	4.71	2.39	7.02	0.001
**ARBC_1000**	19.91	20.47	1.37					
**BRBC_2000**	28.3	31.64	2.12	**BRBC_2000-ARBC_2000**	5.31	1.77	8.85	0.003
**ARBC_2000**	22.99	22.26	1.49					
**BRBC_4000**	32.13	25.41	1.7	**BRBC_4000-ARBC_4000**	4.53	2.35	6.71	0.001
**ARBC_4000**	27.6	24.75	1.66					
**BLAC_250**	38.52	28.94	1.94	**BLAC_250-ALAC_250**	6.70	4.08	9.33	0.001
**ALAC_250**	31.82	26.25	1.76					
**BLAC_500**	37.13	30.52	2.04	**BLAC_500-ALAC_500**	5.00	2.52	7.48	0.001
**ALAC_500**	32.13	27.65	1.85					
**BLAC_1000**	36.88	31.49	2.11	**BLAC_1000-ALAC_1000**	4.48	1.98	6.97	0.001
**ALAC_1000**	32.41	29.03	1.94					
**BLAC_2000**	39.3	36.14	2.42	**BLAC_2000-ALAC_2000**	4.27	0.98	7.57	0.011
**ALAC_2000**	35.02	30.79	2.06					
**BLAC_4000**	46.91	31.79	2.13	**BLAC_4000-ALAC_4000**	5.13	2.91	7.36	0.001
**ALAC_4000**	41.77	32.46	2.17					
**BLAC_6000**	49.39	33.23	2.23	**BLAC_6000-ALAC_6000**	5.25	2.86	7.63	0.001
**ALAC_6000**	44.15	34.2	2.29					
**BLBC_500**	27.31	22.46	1.5	**BLBC_500-ALBC_500**	3.88	1.75	6.01	0.001
**ALBC_500**	23.43	20.48	1.37					
**BLBC_1000**	26.41	22.89	1.53	**BLBC_1000-ALBC_1000**	2.42	0.38	4.45	0.02
**ALBC_1000**	24	22.04	1.48					
**BLBC_2000**	30.13	23.79	1.59	**BLBC_2000-ALBC_2000**	2.24	0.43	4.05	0.016
**ALBC_2000**	27.89	23.96	1.6					
**BLBC_4000**	36.01	25.17	1.69	**BLBC_4000-ALBC_4000**	3.41	1.55	5.27	0.001
**ALBC_4000**	32.6	26.11	1.75					

[BRAC: Before Right Ear Air Conduction = Right ear air conduction (pre-treatment); BRAC average: Air conduction average of hearing intensities at 500/1000/2000/4000 frequencies; ARAC: After Right Ear Air Conduction = Right ear air conduction (post-treatment); ARAC average: ARAC—average air conduction hearing thresholds at 500/1000/2000/4000 frequencies; BLAC: Before Left Ear Air Conduction = Left ear air conduction (pre-treatment); BLAC average: BLAC—average air conduction hearing thresholds at 500/1000/2000/4000 frequencies; ALAC: After Left Ear Air Conduction = Left ear air conduction (post-treatment); ALAC average: ALAC—average air conduction hearing thresholds at 500/1000/2000/4000 frequencies].

**Table 4:** Relationship between pre- and post-treatment averages of hearing levels and pre-treatment grade and post-treatment score.

	BRCA	ARAC	BLAC	ALAC	Pre-Treatment Grade
**ARAC**	0.770 **	1			
**BLAC**	−0.149 *	0.03	1		
**ALAC**	−0.06	0.126	0.847 **	1	
**Pre-treatment Grade**	0.542 **	0.418 **	0.528 **	0.457 **	1
**Post-treatment Score**	0.345 **	0.589 **	0.499 **	0.658 **	0.586 **

[BRAC average: BRAC—air conduction average of hearing thresholds at 500/1000/2000/4000 Hz; ARAC average: ARAC—air conduction average of hearing thresholds at 500/1000/2000/4000 Hz; BLAC average: BLAC—air conduction average of hearing thresholds at 500/1000/2000/4000 frequencies, ALAC average: ALAC—air conduction average of hearing thresholds at 500/1000/2000/4000 frequencies), * *p* < 0.05; ** *p* < 0.01].

**Table 5:** Average air pollution levels over the last 9 years.

	Mean	STD	Minimum	Maximum	National Limit	WHO Limit	EU Limit
**PM_10_ [µg/m^3^]**	50.86	16.63	22.72	112.38	70	50	50
**SO_2_ [µg/m^3^]**	14.03	13.62	1.54	92.52	410	500	350
**PM_2.5_ [µg/m^3^]**	25.86	11.23	11.08	67.92	15	15	
**NOx [µg/m^3^]**	116.86	36.18	46.95	234.80			
**NO [µg/m^3^]**	42.26	23.40	5.99	124.74			
**CO [µg/m^3^]**	1073.98	448.64	321.04	3053.38	10,000	4000	10,000
**NO_2_ [µg/m^3^]**	75.40	18.89	35.92	127.32	270	200	200

[SO_2_: Sulphur dioxide; NO_2_: nitrogen dioxide; CO: carbon monoxide; O_3_: Ozone; PM_10_: Particulate matter].

**Table 6:** Cumulative risk and air parameters by year.

	Mean Square	F	Sig.
**Cumulative Risk**	171,930.99	11.354	0.001
PM_10_ [µg/m^3^]	216.489	7.843	0.001
SO_2_ [µg/m^3^]	148.933	7.006	0.001
PM_2.5_ [µg/m^3^]	99.852	7.484	0.001
NOx [µg/m^3^]	840.632	14.741	0.001
NO [µg/m^3^]	388.001	11.146	0.001
CO [µg/m^3^]	137,730.16	12.381	0.001
NO_2_ [µg/m^3^]	198.78	20.603	0.001

[SO_2_: Sulphur dioxide; NO_2_: nitrogen dioxide; CO: carbon monoxide; O_3_: Ozone; PM_10_: Particulate matter].

## Data Availability

The data sets used during the current study are available from the corresponding author on reasonable request.

## Abbreviations

**ALAC**	After Left Ear Air Conduction = Left ear air conduction (post-treatment)
**ARAC**	After Right Ear Air Conduction = Right ear air conduction (post-treatment)
**AQI**	The National Air Quality Index
**BLAC**	Before Left Ear Air Conduction = Left ear air conduction (pre-treatment)
**BRAC**	Before Right Ear Air Conduction = Right ear air conduction (pre-treatment)
**CO**	Carbon monoxide
**NO_2_**	Nitrogen dioxide
**O_3_**	Ozone
**PM_10_**	Particulate matter
**SPSS**	Statistical Package for the Social Sciences
**SSHL**	Sudden sensorineural hearing loss
**SO_2_**	Sulphur dioxide
